# Digital anatomical measurements and crucial bending areas of the fixation route along the inferior border of the arcuate line for pelvic and acetabular fractures

**DOI:** 10.1186/s12891-016-0974-2

**Published:** 2016-03-15

**Authors:** Chun Bi, Xiaoxi Ji, Fang Wang, Dongmei Wang, Qiugen Wang

**Affiliations:** Trauma Center, Shanghai General Hospital, School of Medicine, Shanghai Jiaotong University, 650 Xin Songjiang Road, Shanghai, 201620 P. R. China; School of Mechanical Engineering, Shanghai Jiaotong University, Shanghai, P. R. China

**Keywords:** Digital measurement, Inferior border of Arcuate line, Fractures of pelvis and acetabulum

## Abstract

**Background:**

Better understanding of three-dimensional (3D) morphology of the pelvis at the area of inferior border of the arcuate line is very important, which could guide the surgeons to treat pelvic and acetabular fractures more efficiently. The objective of this study is to provide references for screw placement and design of anatomical internal fixators for the fixation route along the pelvic inferior border of the arcuate line.

**Methods:**

Seventy five cases of computed tomography (CT) scan data were collected using Medical Image Database in Shanghai General Hospital between December 2009 and November 2010. 44 males and 31 females, aging from 21 to 91 years (average: 57.8 years) were enrolled. Using MIMICS 13.0, these data were used for three dimensional (3D) reconstructions of pelvic model. A curve from the pubic tubercle, along the inferior border of the arcuate line, to the sacroiliac joint was depicted and then divided into 11 equal parts. The measurements of whole length of the curve, the radius of the curvature and the thickness of bone at each decile point were performed, respectively.

**Results:**

The thinnest bone thickness at acetabular area was 17.24 ± 2.90 mm and 9.94 ± 2.69 mm for male and female, respectively. The radius of curvature at the decile points 1, 8 and 10 were smaller compared with the surrounding points.

**Conclusions:**

Using a screw shorter than 10 mm perpendicular to the bone surface along the inferior border of the arcuate line can avoid intra-articular screw penetration. There should be more recontouring of the plate at the areas of pubic tubercle and posterior edge of the acetabulum when placing a fixator along this fixation route. This study provides solid guidance for pelvic and acetabular surgeries as well as designing of anatomical fixators along inferior border fixation route at this area.

## Background

Fractures of pelvis and acetabulum are usually high-energy injuries, which often associated with multiple organ injuries that cause high morbidity and mortality [1]. Because the anatomy, diagnosis, classification, reduction and fixation are relatively complex and difficult, great challenges still exist for treating pelvic and acetabular fractures. With the deeper apprehension of pelvic and acetabular fractures in recent years, there is a tendency for managing these fractures surgically to achieve anatomical reduction [2–5]. The anterior approaches, such as the ilioinguinal and Stoppa approaches, are always employed to treat pelvic and acetabular fractures [6].

In 1961, Letournel first elaborated the classical ilioinguinal approach treating for the fractures of acetabulum [4]. Stable fixation could be achieved by placing a plate along the superior border of the arcuate line through this approach. However, difficulties for reduction and fixator placement with this approach at the area of the quadrilateral surface limit its application. The risk of blood vessels injuries, especially the Corona Mortis, is also a major concern of this approach [7, 8]. The Stoppa approach allows a wide view of the inner surface of the pelvis, which could provide direct vision of the quadrilateral surface, the anterior column and the arcuate line [9]. Internal fixators are relatively easier to be placed inferior to the arcuate line through this approach. The plate could be placed from the front of the pubic tubercle, the inner side of pubic branch, the inferior border of arcuate line edge, to the sacroiliac joint. It is a perfect fixation route for placing the plate to restore the integrity of the pelvis since it could offer a relative regular and smooth surface with adequate bone thickness [10–12].

In consideration of the totally different and more complicated anatomical morphology of the pelvic and acetabular surface compared with the long bone, only the completely matching between the plate and the bone surface can get the excellent reduction. Nevertheless, repeated retouring and adjusting the plate would obviously prolong the operation time. Meanwhile, it would damage the distribution of screw holes and the strength of the plate during the recontouring procedure which would also bring the limited reduction potential and the incidences of complications in future [13–15].

In order to recontour the plate more efficiently during the surgery and avoid potential complications as well, a better understanding of three-dimensional (3D) morphology of the pelvis along the inferior border of the arcuate line is very important. Nevertheless, there is not enough data about this anatomical site to provide insight for clinical practice currently. Previous publication have reported the parameters for plate bending and crucial bending points at the superior border of the arcuate line, which were helpful for pelvic and acetabular surgeries [16]. The approach via the superior border of the arcuate line provides exposure to the anterior wall, anterior column while via the inferior border it could better expose the above-mentioned structures and quadrilateral surface as well as avoid damaging the dangerous Corona Mortis. Due to these differences, the objective of this retrospective observational study is to acquire essential parameters for the description of bone thickness and anatomical shape along the fixation route from the pubic tubercle to the sacroiliac joint at the area of inferior border of the arcuate line and to provide solid guidance for pelvic and acetabular surgeries as well as design of fixators.

## Methods

### Patients and three-dimensional (3-D) reconstruction

Seventy five patients (44 men, 31 women with the mean age of 57.8 years, range: 29–91 years, Table 1), from December 2009 to November 2010, diagnosed with lower limb varicose veins without injuries of acetabulum and pelvis were collected in Shanghai General Hospital.

**Table 1:** Age distribution of all 75 patients

	21–30 year	31–40 year	41–50 year	51–60 year	61–70 year	71–80 year	≥81 year
Male	1	2	5	9	13	7	7
Female	0	3	4	8	9	5	2
Total	1	5	9	17	22	12	9

**Fig. 1:**
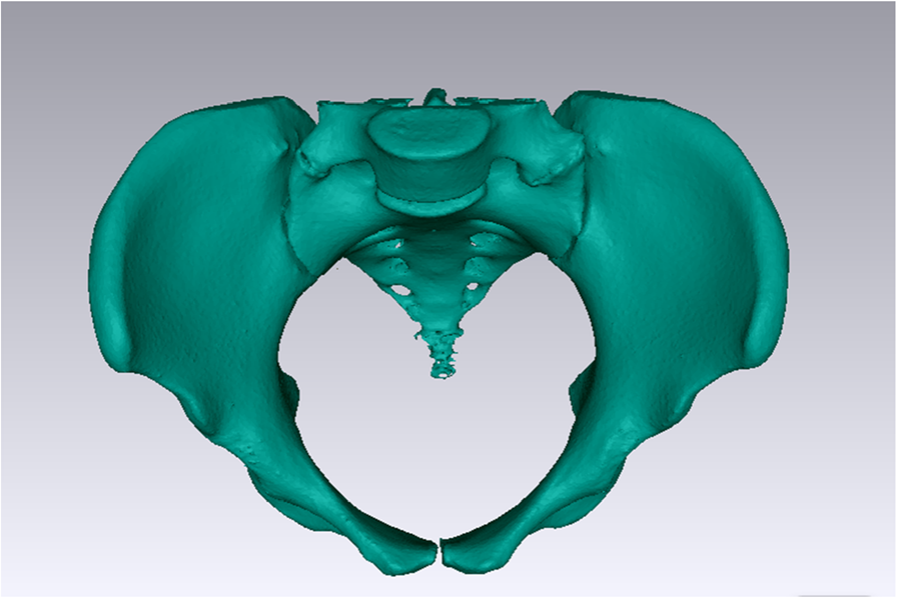
The three-dimension reconstruction of pelvis. Each patient’s raw imaging data were saved with DICOM format then imported to Mimics 13.0, by which 3-D model of pelvis was reconstructed

A 64-channel CT (GE, US) with an acquisition thickness slice of 0.75 mm at 0.2-s intervals for imaging of the pelvis was used for the scanning of these patients. Each patient’s raw imaging data were saved with DICOM format then imported to Mimics 13.0(Leuven, Belgium). Via Mimics 13.0, 3-D model of pelvis was reconstructed (Fig. 1). All obtained 3-D models were processed by thresholding segmentation, region growing, surface smoothing, and stored with STL format. The further smoothing and noise reduction were performed by the Geomagics 10.0 software (Geomagic, US) after the mesh model with STL format was imported.

### Measurements

In order to depict the space curve at the cortical surface, several points were chosen along with the route from the pubic tubercle, the pubic ramus inner side, arcuate line, to sacroiliac joint. All these points were located at the cortical surface below the pelvic brim with the distance of 10 mm.10 mm used as the distance from the measurement point to the pelvic brim, was a commonly used screw insertion point at this area.

**Fig. 2:**
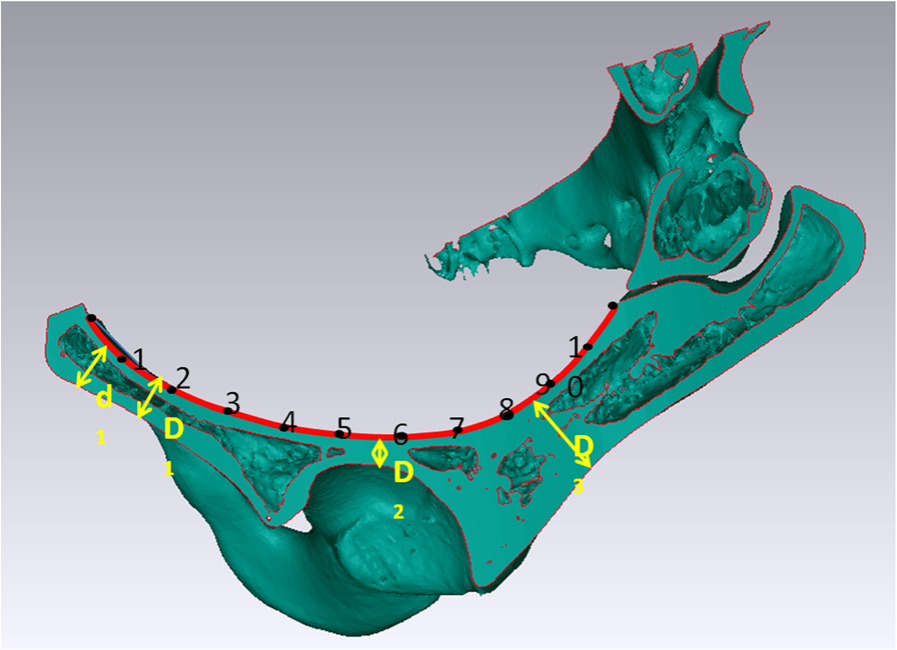
The digital measurements of the inferior border of the arcuate line. By dividing the curve into eleven equal parts, the radius of curvature at each decile point, r1,r2, …,r10, and the bone thickness of each decile point, d1,d2, …,d10, were achieved from front to back. The measurement of thinnest bone area at each part, D1, D2, D3 was performed

By means of intersecting extrusion, the model for measurement and the curve were acquired, and then imported to the software UG (Unigraphics NX) where the measurement for the curve’s whole length (L) can be performed. Eleven equivalent parts of the curve from pubic tubercle to sacroiliac joint were obtained by sectioning the curve at decile points. Both the radius of curvature at these points, r1, r2, …, r10 and the thickness of bone at these points, d1, d2, …, d10 were measured, respectively. With different anatomical area of acetabulum, three parts named front-acetabular, acetabular, and post-acetabular areas, respectively, were achieved by sectioning this curve. Then, the measurement of thinnest bone area at each part, D1, D2, D3 was performed (Fig. 2).

### Statistical analysis

Statistical analysis was performed using the descriptive methods of SPSS v.19 and all data were given as mean ± standard deviation. The comparison of the radius of curvature at different decile point was evaluated by One-way ANOVA with a post hoc test. *P* < 0.05 was considered significant.

## Results

From Tables 2 and 3, the radius and the bone thickness of curvature at each decile point can be summarized. The thinnest bone thicknesses of the front-acetabular, acetabular, and post-acetabular areas were 12.59 ± 2.88 mm、12.52 ± 3.54 mm、24.17 ± 3.25 mm, respectively (Table 4). Significant differences were found for thinnest bone thicknesses of these three areas between male and female.

**Table 2:** The radius of curvature at each decile point

	r_1_	r_2_	r_3_	r_4_	r_5_	r_6_	r_7_	r_8_	r_9_	r_10_
Total (mm)	43.00 ± 20.52	57.66 ± 19.34	76.16 ± 24.64	88.90 ± 28.63	89.63 ± 36.74	8942 ± 34.68	70.33 ± 28.78	43.35 ± 11.31	45.25 ± 18.24	42.90 ± 23.21
Male (mm)	56.48 ± 19.11	70.27 ± 18.42	96.56 ± 23.94	121.63 ± 32.68	125.78 ± 35.98	114.32 ± 31.77	97.30 ± 27.82	55.07 ± 12.58	60.73 ± 17.76	56.78 ± 19.50
Female (mm)	50.99 ± 20.06	65.98 ± 17.76	80.88 ± 25.17	88.81 ± 20.55	89.37 ± 38.62	99.11 ± 36.83	71.49 ± 30.48	44.55 ± 9.33	48.43 ± 18.73	51.43 ± 26.00
*P* value	0.004	0.002	0.167	0.983	0.963	0.057	0.773	0.443	0.213	0.010

**Table 3:** The bone thickness at each decile point

	d_1_	d_2_	d_3_	d_4_	d_5_	d_6_	d_7_	d_8_	d_9_	d_10_
Total (mm)	15.47 ± 3.88	13.28 ± 3.21	15.65 ± 4.04	23.01 ± 5.23	19.93 ± 5.84	13.19 ± 3.61	17.43 ± 4.39	32.52 ± 8.55	28.25 ± 5.91	24.69 ± 3.07
Male (mm)	20.57 ± 3.58	17.59 ± 2.57	21.45 ± 3.35	30.05 ± 3.84	27.25 ± 6.02	18.06 ± 2.87	21.01 ± 3.35	43.65 ± 10.31	36.64 ± 4.93	29.02 ± 2.66
Female (mm)	13.31 ± 3.25	10.79 ± 2.26	12.18 ± 1.74	18.48 ± 3.18	18.08 ± 5.11	10.36 ± 2.47	17.10 ± 5.59	31.35 ± 5.05	23.34 ± 2.96	22.33 ± 1.79
*P* Value	<0.001	<0.001	<0.001	<0.001	0.02	<0.001	0.589	0.274	<0.001	<0.001

**Table 4:** The thinnest bone thickness and the whole curvature length: The thinnest bone thickness of the front-acetabular, acetabular, and post-acetabular areas, the whole length of the curvature

	D_1_	D_2_	D_3_	L
Total (mm)	12.59 ± 2.88	12.52 ± 3.54	24.17 ± 3.25	129.09 ± 10.24
Male (mm)	16.35 ± 1.99	17.24 ± 2.90	28.67 ± 2.68	133.88 ± 9.30
Female (mm)	10.08 ± 1.92	9.94 ± 2.69	21.57 ± 2.00	135.50 ± 7.89
*P* Value	<0.001	<0.001	<0.001	<0.001

The mean value of the radius of curvature at decile points 1, 8 and 10 were smaller compared to their adjacent points 2, 7 and 9, 10, respectively. There were statistically significant differences for radius of curvature between the decile points 1 and 2, 7 and 8, 8 and 9, 9 and 10 (*P* < 0.05) were found (Table 5).

**Table 5:** Comparisons of different decile points: The significance test of difference between the mean values of the radius of curvature at decile points 1 and 2; 7 and 8; 8 and 9; 9 and 10

	Curvature of radius			
	Point1 vs. Point2	Point7 vs. Point8	Point8 vs. Point9	Point9 vs. Point10
*P* Value	<0.001	<0.001	<0.001	<0.001

## Discussion

The Stoppa approach, which is widely used for pelvic and acetabular fractures, provides direct vision to the quadrilateral surface, the anterior column and the arcuate line [9]. Meanwhile, it can avoid injuring dangerous blood vessels specially Corona Mortis which can be easily injured during exposure of the superior border of the arcuate line via the ilioinguinal approach [7, 8]. Another advantage of the inferior border approach is it gives a better and direct exposure for assessment and treatment of quadrilateral area fractures [9]. In the treatment of the peri-anterior column fractures and rotationally unstable pelvic fractures, the area of the inferior border of the arcuate line with its forward extension provides an ideal area for placing the fixator [17]. This is a perfect fixation area for placing the plate to restore the integrity of the pelvis since it offers a relative regular and smooth surface with adequate bone thickness and clearly surgical expose as well. The anterior wall, anterior column, and especially the fracture with quadrilateral surface can be fixed by placing the reconstruction plate along this fixation route [18].

In consideration of the totally different and more complicated anatomical morphology of the pelvic and acetabular surface compared with the long bone, only the completely matching between the plate and the bone surface can get the excellent reduction. However, repeated retouring and adjusting the plate would obviously prolong the operation time. Meanwhile, it would damage the distribution of screw holes and the strength of the plate during the recontouring procedure which would also bring the limited reduction potential and the incidences of complications in future [13–15]. As mentioned above, the better comprehension of the unique anatomical morphology of pelvis is beneficial to measure the essential parameters at this fixation route of inferior border, to discover the key points of the plate-recontouring, and to provide guidance for design of the anatomical internal fixators.

In order to deeply apprehend the crucial turning points as well as the morphology of the cortical surface at this fixation area, the comparison about the radius of curvature between each decile point and those of its adjacent points was performed, respectively. The radius of curvature at each point could be interpreted as the bending degrees during operation. The results of this study could provide references to assure more accurate plate bending, which would be important to reduce the complications. Compared to the adjacent points, the smaller radii of curvature can be found in the1, 8, 10 decile points. From the view of anatomical perspective, this curve has a relatively greater bending degree at the areas of the pubic tubercle and the posterior edge of the acetabulum. Thus, there should be corresponding anatomical areas to the larger degree of bending in this curve at the pubic tubercle and the posterior edge of the acetabulum. To learn how to accurately recontour the plate and better match the bone surface is not an overlooked factor that has effect on the prolonged learning curve of the pelvic and acetabular surgeries [19, 20]. According to our results, the plate should be bent more at the pubic tubercle and posterior edge of acetabulum during the surgeries for acetabulum and pelvis fracture fixation.

During the fixation of pelvic and acetabular fractures, since the articular surface cannot be directly observed, the screw might penetrate into the hip joint which frequently leads to malunion and traumatic osteoarthritis in future [21]. Several methods, such as intra-operative radiographs and fluoroscopy, have been reported to prevent this complication. However, these methods inevitable increase the operation time and bring the potential risk of trauma [22, 23]. In this study, with the purpose of visualized measurements on the exposed cross-section at anterior column and measuring the thinnest cortical thickness at acetabulum, the Geomagics was used to transect cortical surface of this area perpendicularly. To avoid damaging the blood vessel and nerve at the greatest extent, the bone thickness of each decile point taken as reference for choosing proper length of the screw during the operation was measured, respectively. Meanwhile, the safe and effective screw placement can be acquired from the thinnest diameter of the bone in acetabulum to prevent intra-articular screw penetration of the hip joint. As the results showed, placing a screw, shorter than 17 mm and 10 mm for male and female, respectively, perpendicular to the cortical surface along the above-mentioned inferior border fixation route would prevent intra-articular screw penetration. These will make contributions in perfect plate-recontouring. Moreover, these essential parameters we gathered can help making the internal fixators more accurately. Although there is still room for improvement, this study proposed practical guidance for plate-screw fixation in this area.

### Limitations

There are some limitations for this study. All samples were enrolled at the authors’ hospital, it would be more meaningful to perform multi-center investigations for comparison. The complete data of these sample’s height and weight were not gathered because of the different medical conditions of each sample. In addition, the average age of current study was relatively old, younger samples would be considered in future studies.

## Conclusion

The current study acquired essential parameters which describe the thickness of bone and anatomical shape from the pubic tubercle to the sacroiliac joint at the area of inferior border of the arcuate line. Using a screw shorter than 10 mm perpendicular to the bone surface along the abovementioned fixation route would not have risk of intra-articular screw penetration during the internal fixation of the fractures fragment. There should be more recontouring of the plate at the areas of the pubic tubercle and the posterior edge of the acetabulum when placing a fixator along this route. This 3D measurement study provides better guidelines for the pelvic and acetabular surgery as well as designing anatomical internal fixators.
